# Unleashing nature's potential and limitations: Exploring molecular targeted pathways and safe alternatives for the treatment of multiple sclerosis (Review)

**DOI:** 10.3892/mi.2023.102

**Published:** 2023-08-17

**Authors:** Sara Fathallah, Ahmed Abdellatif, Mona Kamal Saadeldin

**Affiliations:** 1 Biotechnology Program, School of Science and Engineering, American University in Cairo, New Cairo 11835, Egypt; 2 Biology Department, School of Science and Engineering, American University in Cairo, New Cairo 11835, Egypt; 3 Department of Chemical and Biomolecular Engineering, University of Notre Dame, Notre Dame, IN 46556, USA

**Keywords:** multiple sclerosis, molecular pathways, therapeutic approaches, naturally occurring molecules, antioxidants, neurodegenerative diseases

## Abstract

Driven by the limitations and obstacles of the available approaches and medications for multiple sclerosis (MS) that still cannot treat the disease, but only aid in accelerating the recovery from its attacks, the use of naturally occurring molecules as a potentially safe and effective treatment for MS is being explored in model organisms. MS is a devastating disease involving the brain and spinal cord, and its symptoms vary widely. Multiple molecular pathways are involved in the pathogenesis of the disease. The present review showcases the recent advancements in harnessing nature's resources to combat MS. By deciphering the molecular pathways involved in the pathogenesis of the disease, a wealth of potential therapeutic agents is uncovered that may revolutionize the treatment of MS. Thus, a new hope can be envisioned in the future, aiming at paving the way toward identifying novel safe alternatives to improve the lives of patients with MS.

## 1. Introduction

Multiple sclerosis (MS) is a prevalent autoimmune neurodegenerative disorder affecting over 2.5 million individuals globally according to the Atlas of MS (1). In MS, B-cells and T-cells infiltrates the blood-brain barrier (BBB) and attack the myelin sheath through microglial activation and the destruction of oligodendrocytes. In addition, plasma cells create antibodies targeting the myelin sheath, leading to a variety of non-distinctive symptoms, such as optic neuritis, tingling sensations, muscle weakness, anxiety, depression and cognitive impairment. As a chronic progressive disease, the symptoms of MS worsen over time, often leading to disability within 10-20 years (2).

The diagnosis of MS in its early stages is challenging due to the non-specific symptoms. Neurologists typically use magnetic resonance imaging scans of the brain and spinal cord, along with blood tests for vitamin D and blood cells to exclude other illnesses and confirm the diagnosis of MS (3). The present review discusses the different MS categories, molecular pathways involved in pathogenesis, and natural therapies as potential references for alleviating the symptoms of MS.

## 2. Categories of multiple sclerosis

MS can be categorized into four types based on the clinical course of the disease, as follows: i) Relapsing-remitting MS (RRMS): This type is common among young individuals characterized by attacks followed by partial or complete remission in the early stages of the disease. However, with multiple attacks, the nerves deteriorate and the damage cannot be fully reversed. ii) Primary progressive MS (PPMS): The symptoms in this type worsen without remissions, with occasional plateau periods. iii) Secondary progressive MS (SPMS): This type follows RRMS, but with more severe symptoms. iv) Progressive relapsing MS (PRMS): In this type, symptoms worsen from the beginning with periods of remission (4,5).

The frequency and intensity of the attacks are influenced by lifestyle factors. Smoking worsens symptoms and accelerates disability progression compared to non-smokers (6). Stress, poor diet and a lack of sleep can also aggravate MS; thus, adopting a healthy lifestyle, including quitting smoking and regular exercise, may help alleviate some of the symptoms (7).

## 3. Molecular targeted pathways for therapy

MS is a multi-causation disease model with multiple contributing factors. Thus, the present review summarizes the main pathways involved in the development of MS and the potential available therapeutic targets. Understanding these molecular pathways is essential for developing targeted therapies to alleviate morbidity and mortality associated with MS.

### Toll-like receptor (TLR)/myeloid differentiation factor 88 (MyD88) pathway. TLR/MyD88 overview

One of the key pathways involved in MS is the TLR/MyD88 pathway. TLRs are members of the pattern recognition receptor family and play a crucial role in both innate and adaptive immune responses by identifying microbes and abnormal cells (8). There are a minimum of 10 TLR members; their activation leads to the activation of adaptive immunity and triggers a series of inflammatory consequences, primarily through the MYD88-mediated signaling pathway (9).

TLRs, particularly TLR2 and TLR4, are expressed at higher levels in patients with MS and in rodents with experimental autoimmune encephalomyelitis (EAE), an animal model of MS, indicating their role in the pathogenesis of MS (10-12). Further information regarding the importance of the EAE animal model can be found in the studies by Constantinescu *et al* (13) and Mahmoudi *et al* (14). In addition, animal experiments with TLR2-deficient animals have demonstrated increased remyelination, suggesting the involvement of TLR2 in oligodendrocyte precursor cell development (10). Another TLR2/4 ligand, HMGB-1, is present in active MS and EAE lesions and acts as a powerful proinflammatory signal by interacting with TLR2 or TLR4 (10,15).

Similarly, the deletion of TLR4 in CD4^+^ T-cells in EAE models significantly alleviates clinical symptoms and inhibits the occurrence of EAE, mainly by reducing Th17 and, to a lesser extent, Th1 responses (11,16). Moreover, the TLR4 signaling pathway also influences interferon (IFN)-β responsiveness in patients with MS where IFN responders exhibit a significantly lower baseline expression of the TLR4-negative regulator, interleukin (IL)-1 receptor-associated kinase (IRAK)3, compared to non-responders, resulting in proving the critical roles of TLR2 or TLR4 incorrect responses in MS etiology (17).

The TLR-MyD88 signaling pathway plays a crucial role in the onset and progression of autoimmune diseases, including MS and EAE. It controls dendritic cell (DC) antigen presentation, BBB integrity, and T- and B-cell activation (18). MyD88 functions as a downstream adaptor for TLR family members and IL-1R-associated kinase, leading to the activation of NF-κB, mitogen-activated protein kinase (MAPK), and activator protein 1 pathways, which trigger the production of pro-inflammatory cytokines, such as IL-1β, IL-6 and TNF-α. IL-1β activation in neurons depends on two signaling pathways; a MyD88-dependent pathway that causes inflammation and a neuroprotective MyD88-independent pathway. MyD88 also influences the differentiation of splenic Th17 cells and the expression of IL-6 and IL-23p40 by splenic monocyte-derived DCs. Additionally, MyD88 activation promotes the expression of CD40^+^, CD80^+^, CD86^+^ and major histocompatibility complex class II in immature antigen-presenting cells (19-21). These findings highlight the significance of the TLR-MyD88 pathway in MS pathogenesis and suggest it as a potential therapeutic target.

*TLR/MyD88 as a therapeutic target.* Previous studies have demonstrated the involvement of TLRs in the pathogenesis of MS, rendering TLR-MyD88-targeted immunotherapy a potential approach for the treatment of MS and other inflammatory diseases. TLR antagonists, including T2.5 and OPN-305 for TLR2, and IMO-3100 for both TLR7 and TLR9, have been developed, and their efficiency and selectivity are being continuously improved (22-25).

These TLR/MyD88 signaling pathway inhibitors function through various mechanisms, including binding to TLR domains to compete with TLR agonists (e.g., dsRNA, and its synthetic analogs, such as ligand Poly (I:C) (26), inhibiting signal transmission, promoting axon sparing and encouraging oligodendrocyte progenitor cell invasion for myelin repair. Soluble TLRs can also prevent TLRs from functioning normally by blocking TLR activators, such as lipopolysaccharide (LPS) and the human immunodeficiency virus (HIV) (24,27,28).

The inhibition of MyD88 is particularly promising, as it is essential for most TLRs (except TLR3 and a portion of TLR4). For instance, the short version of MyD88 (sMyD88) can diminish TLR-MyD88 signaling by blocking IRAK1 phosphorylation. Additionally, BB-loop decoy can interfere with the MyD88 Toll/IL-1 resistance domain or the full-length MyD88, thereby preventing the TLR-MyD88 signaling pathway from functioning (28). These therapeutic approaches hold the potential for impacting MS progression positively.

### NF-κB pathway. NF-κB pathway overview

NF-κB is a crucial transcription factor in the inflammatory signaling cascade. Its activation occurs upon IκBα phosphorylation followed by ubiquitination and degradation. This triggers the translocation of the p50 and p65 NF-κB subunits to the nucleus, stimulating the expression of pro-inflammatory cytokines (29). In patients with MS, the NF-κB pathway is activated, leading to increased pro-inflammatory cytokine expression in brain tissues, particularly in microglia, hypertrophic astrocytes and lymphocytes within active demyelinating plaques (30,31). Functional variants near NF-κB signaling genes in regulatory regions have been identified in patients with MS, enhancing the expression of the NF-κB signaling genes (32).

Th1 and Th17 cells play critical roles in the pathogenesis of MS and are used to differentiate between the different clinical MS phenotypes (33). Th1 cells secrete cytokines involved in cellular immunity and autoimmune diseases, while Th17 cells are primarily responsible for the development of MS and EAE. Both Th1 and Th17 responses are associated with NF-κB activation. IL-12 (34-37) and IL-18 are crucial cytokines involved in Th1 responses, and NF-κB plays a role in their production (38-44). IL-17A is produced by Th17 cells, and IL-23 induces their formation through NF-κB-dependent pathways (45-49).

Th17 cell production can be enhanced by IL-1β. In the cerebrospinal fluid (CSF) of patients with MS, there is an elevated expression of IL-1β, IL-1 receptor accessory protein, and IL-1 receptor antagonist (IL-1Ra). Since IL-1Ra suppresses IL-1 activities, the increased production of this molecule may appear contradictory; however, it has been proposed that it is a component of a defensive mechanism that may be implicated in MS remissions. It has been demonstrated that NF-κB signaling is necessary for IL-1 to encourage T-cells to produce IL-17 (50-52).

It is critical for comprehension to clarify the function of the effector cytokines that are generated by Th17 cells in the induction of EAE. Astrocytes release chemokines and pro-inflammatory cytokines in response to IL-17, which attract leukocytes and is mediated by NF-κB (53). While Th17 cells produce IL-17A and IL-17F, their impact on EAE development is limited as IL-23 also promotes EAE through both IL-17-dependent and independent mechanisms (34,54,55). IL-21 and IL-22 produced by Th17 cells, have been suggested as alternative mediators; however, their direct connection to EAE remains unclear (34,56,57). Understanding these cytokines and their interactions is crucial for the understanding of the pathogenesis of MS and for the identification of potential therapeutic targets.

In summary, the NF-κB pathway and its association with Th1 and Th17 responses play significant roles in the pathogenesis of MS. Targeting this pathway and understanding the roles of specific cytokines may lead to the development of promising therapeutic strategies for MS.

*NF-κB pathway as a therapeutic target*. The NF-κB pathway plays a crucial role in the development of MS with multiple signaling mechanisms involved. While NF-κB modulation is a potential therapeutic target, the most effective approach between direct inhibition and targeting factors regulating NF-κB signaling warrants further investigation. In novel MS treatments, fine-tuning NF-κB signaling by inhibiting the factors that regulate it can restore its balance and offer promising therapeutic possibilities (58). Some novel MS treatments involving the NF-κB signaling pathway are summarized below.

*i) Fingolimod*. Fingolimod, a sphingosine analog, is a novel oral treatment for MS that regulates the NF-κB signaling pathway. It activates the sphingosine kinase 1 (SPHK1) through stimuli such as pro-inflammatory cytokines, including TNF-α, platelet-derived growth factor, VEGF and EGF, which promotes the translocation of SPHK1 to the plasma membrane to phosphorylate sphingosine and produce sphingosine-1 phosphate (S1P) (59-61). S1P is essential for activating the canonical NF-κB signaling pathway through TNF receptor-associated factor 2 and receptor-interacting protein 1. In addition, S1P activates ERK1/2, which in turn leads to an increase in NF-κB production (62-64).

Additionally, fingolimod affects lymphocyte migration by acting on the S1P1 receptor, affecting T- and B-cell movement and accumulation in lymphoid organs (65-67). By blocking immune cell trafficking, fingolimod prevents immune cells entry into the central nervous system (CNS), and reduces neuroinflammation and neurodegeneration. Fingolimod also crosses the BBB, allowing it to act centrally on astrocytes. By inhibiting astrocyte activation, fingolimod exerts a neuroprotective effect, encouraging a CNS reparative process (60,68).

In conclusion, the regulation of the NF-κB pathway by fingolimod and its effects on immune cell migration and astrocyte activation render it a promising therapeutic option for MS, potentially attenuating disease progression and protecting against neurodegeneration.

ii) Teriflunomide. Teriflunomide is an active metabolite of the anti-rheumatic medication, leflunomide (LN). It inhibits dihydroorotate dehydrogenase, a crucial enzyme for *de novo* pyrimidine production in rapidly growing lymphocytes, leading to cytostatic effects on proliferating B- and T-cells. LN also inhibits TNF-α induced NF-κB activation and other stimulants that activate NF-κB. It further affects the activation of the lymphoid cell kinase (p56lck), an Src family member, and the canonical Raf-MAPK pathways, thus reducing NF-κB synthesis (68-70). These mechanisms contribute to its therapeutic effects on MS.

*iii) IL-17*. IL-17A, produced by Th17 cells, plays a crucial role in human autoimmune, inflammatory and neurodegenerative diseases. It is produced by various immune cells, such as CD8^+^ (Tc17) cells, ‘natural’ Th17 cells, group 3 innate lymphoid cells and natural killer T-cells, as well as CNS resident cells, such as the microglia (71-75). IL-17A contributes to neuroinflammation in disorders, including Parkinson's disease (PD), Alzheimer's disease (AD) and amyotrophic lateral sclerosis. Patients with MS, AD and PD have considerably higher levels of IL-17A in their CSF and plasma, and IL-17A expression levels are associated with the severity and progression of the disorders (74,76-80). IL-17A increases inflammation and it functions more as a regulatory element in the network of generated cytokines than as a direct mediator of tissue injury (81-83). Additionally, some studies have reported the effects of medicinal plants (such as resveratrol, matrine, huperzine A and ellagic acid) on IL-17A levels in neurodegenerative disorders (84-88).

Notably, in MS, IL-17A is closely associated with the breakdown of the BBB and neutrophil growth in the CSF (47,89,90). It may also promote glutamate's Ca^2+^-dependent release, contributing to the excitotoxicity of glutamate in MS development. In EAE, there is a substantial increase in IL-17RA expression in the CNS. IL-17RA contributes to the EAE pathophysiology by encouraging CD^4+^ T-cell migration and chemokine secretion after binding to the IL-17RA complex in the CNS (91,92). In addition, microglia secrete numerous soluble substances involved in the CNS immune response, and alterations in microglial cells can result in excessive synapse loss in neurodegenerative disorders (93-95).

IL-17A influences oligodendrocyte lineage cells, preventing their maturation and exacerbating TNF-α-induced oligodendrocyte death. Blocking IL-17A signaling in oligodendrocyte progenitor cells attenuates the severity of EAE, highlighting its role in the progression of MS (96-98). In summary, the involvement of IL-17A in neuroinflammation and its effects on various CNS cells render it a critical target for potential therapeutic interventions in neurodegenerative diseases, particularly in MS.

## 4. Medicinal naturally occurring substances used in the treatment of MS and their mechanisms of action

Nutrition plays a key role in the development of MS, with deficiencies in dietary elements, such as vitamin D and vitamin B12 potentially affecting the progression of MS. The brain tissue is affected by diet through several mechanisms, including mitochondrial dysfunction, epigenetic alterations, gut microbiota and neuroinflammation (99-101). Neurodegeneration manifests itself even in the early stages of MS, and oxidative stress plays a crucial role. In experimental models, oxidative stress has been shown to result in mitochondrial malfunction and the disruption of cell membranes, ultimately causing neuronal cell death (102-104), This has been reviewed by Mao and Reddy (102). However, the presence of antioxidant nutrients in the diet can protect against neuronal or axonal damage, chronic demyelination and oxidative stress. Both oxidative and mitochondrial damage can impair cellular communication predominantly impairing the function of neurons and glial cells (102,105,106).

Both progressive and RRMS involve oxidative damage to myelin and axons caused by inflammatory cytokines, reactive oxygen species (ROS) and phagocytes. Oxidative stress contributes to increased inflammation and damages myelin, resulting in cell death. In clinical settings, inflammatory and oxidative stress mediators, such as cytokines IL-1, IL-6, IL-17 and TNF-α, have been linked to the progression of MS (107,108).

Dietary antioxidants have the potential to regulate immune-inflammatory cell activation and reduce it. These antioxidants also play a role in reducing oxidative stress, which in turn can help prevent persistent demyelination and axonal damage. Some naturally available antioxidants, such as astaxanthin (AST), quercetin and anthocyanin (ACN), have shown promise in their ability to counteract oxidative damage and provide protective effects. Incorporating these natural antioxidants into the diet may hold significant benefits for patients with MS. By reducing inflammation, protecting brain tissue and promoting overall health, these medicinal naturally occurring substances can complement conventional treatments and potentially enhance the management of MS. Further research on and the exploration of the mechanisms of action of these substances are vital in understanding their full therapeutic potential in the treatment of MS.

### AST

Scientists have recently devoted considerable attention to the study of AST, a naturally occurring red fat-soluble xanthophyll carotenoid generated by marine microorganisms. It has been demonstrated that AST possesses potent antioxidant characteristics which are 100-fold more potent than vitamin E and 10-fold more potent than other carotenoids, including lutein, canthaxanthin and zeaxanthin. Its anti-inflammatory, antioxidant and anti-apoptotic actions are the major underlying mechanisms for its protective activities (109). Moreover, the protective effects of AST extend to the brain, where it has been shown to protect against primary brain injuries, death of neurons, the disruption of the BBB, cerebral edema and poor neuronal function by reducing brain inflammation. Further studies also demonstrated that AST has anti-lipid peroxidation and anticancer properties (110-112).

Animal models of neurodegenerative diseases have enabled the better understanding of the molecular mechanisms underlying the onset and progression of MS and other severe neurodegenerative disorders. Additionally, as oxidative damage plays a role in the late onset of extensive neuronal death in neurodegenerative diseases and as AST has been shown to exert antioxidant effects, AST has attracted particular interest as a co-treatment for neurodegenerative diseases (113,114). Animal models have been used by scientists to assess the neuroprotective properties of AST owing to its ability to cross the BBB (109,113,115).

To study the process of demyelination and remyelination in rodent models of MS, cuprizone (CPZ) is the most established technique among several approaches that have been proposed for creating an MS animal model. In the CPZ-induced rat model of MS, it was determined that AST ingestion altered demyelination, oligodendrocyte death and muscular weakening (116,117).

In the context of the animal model of CPZ-induced MS, CPZ has been utilized to create a toxic MS model, followed by analyses conducted on brain tissue to investigate the AST neuroprotective properties. The advantage of using the copper chelator, CPZ, to produce a demyelinating model is its highly reproducible demyelination in the brain tissue. The white matter tract in the rat brain, the corpus callosum, exhibits persistent demyelination by the third week of CPZ exposure. Hence, the animals were exposed to 0.6% CPZ daily for 4 weeks to develop MS (118,119). In a rat model of CPZ-induced MS, the AST administration decreased the oligodendrocyte damage and myelin sheath disintegration. Moreover, quantitative polymerase chain reaction and immunohistochemistry revealed that AST reduced demyelination and oligodendrocyte death in the rat model of CPZ-induced MS (120).

In brief, AST has attracted the interest of scientists due to its significant antioxidant properties, surpassing vitamin E and other carotenoids and its protective effects extend to the brain, thus rendering it a potential candidate for co-treatment in neurodegenerative diseases. Animal models in general and the rodent model of CPZ-induced MS in particular, provided useful insight into the neuroprotective functions of AST that may result in preserving myelin and protecting against oligodendrocyte damage.

### Quercetin

Quercetin is a natural flavonoid abundant in various fruits, herbs and vegetables. It has promising biological benefits, including anti-inflammatory, antioxidant and neuroprotective properties. Preclinical and clinical research has indicated that quercetin may be effective in alleviating MS, rheumatoid arthritis, inflammatory bowel disease and systemic lupus erythematosus (121).

The gut flora plays a critical role in transforming quercetin into easily absorbable molecules by synthesizing glycosidases and other enzymes. Several reports have suggested that quercetin has potent anti-inflammatory properties that function primarily by preventing the generation of cytokines, lowering the expression of cyclooxygenase and lipoxygenase, and preserving the integrity of mast cells (122-126). It can also inhibit the activation of key inflammatory signaling pathways, such as NF-κB, p38 MAPK and ERK1/2, leading to the decreased production of pro-inflammatory cytokines, including IL-1β, IL-6 and TNF-α. Studies have demonstrated that quercetin can lower the production of prostaglandin E-2, IL-1β and leukotriene B-4 in diabetic rats, thus facilitating wound healing in rodents (127,128).

Of note, a previous study focused on the aryl hydrocarbon receptor (AhR) as a key player in regulating inflammatory reactions and immune cell modulation through developing tolerance. Quercetin, a natural AhR ligand, was compared to the synthetic ligand, 2,3,7,8-tetrachlorodibenzo-p-dioxin (TCDD) (129). That study found that only quercetin reduced the activation of T-cells and the migration of LPS-matured DCs compared to TCDD. CD83 was downregulated as a result of the binding of its promoter and activated AhR (129). Moreover, a reduction in the levels of the pro-inflammatory cytokine, IL-12p70, was the result of treating LPS-DCs with quercetin. It also resulted in enhancing the expression of various molecules, such as immunoglobulin-like transcript (ILT)-3, ILT-4, ILT-5, and immunoregulatory molecules disabled-2 adaptor protein (Dab2), in addition to the ectonucleotidases, CD39 and CD73(129). Quercetin also induced a tolerogenic phenotype in quercetin-treated maturing DCs, which are important for controlling the immune response and prevention of autoimmune diseases. These results reveal quercetin to be a strong immunomodulatory agent capable of changing the phenotype and activity of human DCs and shifting the immunological balance away from inflammation and towards resolution (129).

DCs, particularly tolerogenic DCs (tDCs), play a crucial role in regulating immune responses and preventing autoimmune diseases (130-132). Immunogenic tDCs are characterized by the production of immunomodulatory molecules, such as the inhibitory receptors, ILT-2, -3, -4 and -5. Furthermore, tDCs express Dab2 and adenosine triphosphate extracellular level regulation-related factors (133-135). It is worth mentioning that in the EAE mouse model of MS, tDCs decrease disease severity. Plant-derived natural aryl hydrocarbon receptor (AhR) ligands, including indole-3-carbinol and indirubin-3'-oxime, similar to quercetin, can induce tDCs and thus may affect the onset and progression of autoimmune disorders (130,136-138).

As plant-derived compounds are a main source of natural AhR ligands, of note, recent findings have demonstrated an association between dietary chemicals and immunity via the activation of AhR. Following activation, AhR translocates from the cytoplasm to the nucleus and binds the aryl hydrocarbon receptor nuclear translocator (ARNT) to create a heterodimer (139-142). The AhR/ARNT complex then interacts with the xenobiotic response element (XRE) in target genes to stimulate the transcription of several genes, including the AhR repressor (AhRR), and the cytochrome P450 xenobiotic metabolizing enzymes, CYP1A1, CYP1A2 and CYP1B1. By contrast, AhR degradation by proteasomes, ligand metabolism by CYP enzymes, and disruption of the AhR/ARNT complex by AhRR all decrease AhR signaling (143).

While the underlying mechanisms of action are mostly understood, the widespread expression of AhR in immune cells supports its crucial role in the immune system in the steady state and during inflammation. High quantities of AhR are expressed by DCs, and these levels are elevated even further in response to TLR ligands. Hence, natural AhR-activating substances found in food can have an immunomodulatory impact, and AhR signaling has been demonstrated to exert tolerogenic effects on DCs. Similarly, previous research has demonstrated an improved capacity of DCs to multiply Foxp^3+^ Tregs following AhR activation (130,143-147). In addition, it has been demonstrated that the activation of AhR by quercetin impairs bone marrow-derived DC (BMDC) maturation, antigen-presenting ability, and cytokine and chemokine production. Moreover, quercetin has been shown to prevent BMDCs from being activated, which attenuates the development of atherosclerosis. In humans, quercetin has been shown to reduce DC activity and differentiation to mediate anti-atherosclerotic effects (133,148,149).

Furthermore, the administration of quercetin was previously found to markedly decrease the amount of pro-inflammatory cytokines released into the DC-T-cell co-culture supernatants, including IL-1β (4.0-fold), IL-6 (3.6-fold), IFN-(2.5-fold), TNF-α (2.0-fold) and IL-12p70 (5.7-fold) (129). When quercetin was not present during co-culture, similar results were obtained. In this case, IL-1β (1.7-fold), IL-6 (2-fold), IFN-(2.9-fold) and TNF-α (5.6-fold) pro-inflammatory cytokine levels were markedly decreased compared to the mock control (129). The mixed lymphocyte reaction observations were consistent with the fact that TCDD had essentially a minimal impact on cytokine secretion. In the supernatants of those co-cultures, a small tendency towards an enhanced production of IL-6, IFN, TNF-α and IL-12p70 was observed. In none of the examined supernatants was the anti-inflammatory cytokine IL-10 found (129).

In summary, the immunomodulatory and anti-inflammatory effects of quercetin render it a promising agent in the treatment of a number of diseases, including autoimmune conditions. Its ability to activate AhR and induce tDCs suggests it is a promising candidate for regulating immune responses and resolving inflammation. In addition, the widespread availability of quercetin in herbs, fruits and vegetables makes it attractive for further research and potential therapeutic solutions.

### ACNs. Overview of ACNs

ACNs are natural phenolic pigments that have established pharmacological activities. They are widely recognized for their potent antioxidant and anti-inflammatory properties, which account for the range of benefits linked to these compounds, including their neuroprotective effects, and the prevention of age-related chronic illnesses including cancer, cardiovascular diseases and ocular diseases (150). ACNs are also antiviral in nature. They can inhibit the reproduction of viruses, including herpes simplex virus, parainfluenza syncytial, HIV, rotavirus and adenovirus. ACNs are produced basically by plants to entice insects to flowers for pollination and herbivorous animals to fruits for seed dispersal as well as to shield plant cells from UV radiation damage. To date, >700 ACNs have been found in nature. Several plant families, including Vitaceae, Rosaceae, Ericaceae, Saxifragaceae, Caprifoliaceae, Cruciferae and Fabaceae include ACNs (150,151).

Several ACNs with neuroprotective properties are found in numerous dietary products. For instance, ACNs found in blue maize have been shown to shield the brain from the mitochondrial DNA common deletion that is caused by moderate ethanol intake. ACN-rich purple corn extract lowers trigeminal macrophage infiltration and microglia activation both *in vivo* and *in vitro* and protects against the development of orofacial allodynia in an *in vivo* model of inflammatory trigeminal pain (152,153). Moreover, purple corn has neuroprotective effects equivalent to the anti-inflammatory properties of acetylsalicylic acid, which do not alter microglial activity (152,153). Thus, to reduce the medication dose for trigeminal pain and subsequently, the side-effects, it may be suggested to use ACN-rich dietary supplements as a co-adjuvant therapy to existing medications or as a preventative approach against neuroinflammatory conditions such as trigeminal pain (150).

ACNs found in food are recognized as being more potent antioxidants than vitamins E and C. They can stimulate antioxidant enzymes, regulate antioxidant defense systems and encourage the formation of glutathione, a key antioxidant molecule. They can also chelate metal ions, such as iron and copper, thereby reducing the production of harmful free radicals by Fenton and other processes. Additionally, ACNs directly increase the enzymatic activity of antioxidant enzymes such as superoxide dismutase, catalase, and glutathione peroxidase (GP), as well as nuclear factor erythroid 2-related factor 2, a protein that plays a key role in cellular defense against oxidative stress (154,155).

Furthermore, ACNs counteract the negative effects of hazardous substances. For instance, ACNs from blueberry extract counteract the effects of acrylamide, a harmful compound formed during cooking, by reducing liver GP depletion and excessive ROS generation. In addition, they have been shown to successfully reduce the activity of CYP2E1 protein, a protein associated with the metabolism of harmful substances, in mice treated with acrylamide (156). The ACN-mediated protection against ethanol- and ROS-mediated damage is also dependent on the suppression of CYP2E1 protein activity; ACNs in *Gynura bicolor* have been shown to restore the glutathione content and decrease the ROS and glutathione disulfide levels in the livers of ethanol-exposed mice (157).

Some of the molecular mechanisms of ACNs. In chronic illnesses and the aging process, oxidative stress occurs when the synthesis of ROS and reactive oxygen-nitrogen species outpaces the antioxidant systems of cells or tissues. This subsequently damages macromolecules (such as proteins, lipids and DNA). The primary defense against infections and injuries is acute inflammation, which is often followed by the resolution of inflammation. A number of chronic illnesses, including cardiovascular and neurological disorders, diabetes mellitus, cancer and neurodegenerative diseases, are considered to be accelerated by the persistent inflammation that results from the lack of resolution (158,159). Some mechanisms of ACNs are the following: i) ACNs cause a decrease in the levels of pro-inflammatory cytokines, such as inducible nitric oxide synthase and cyclooxygenase-2 by inhibiting the MAPK signaling cascade that includes p38, JNK and ERK (160,161); ii) ACNs prevent the activation of NF-κB, a transcription factor that controls numerous genes involved in the inflammatory response; this response involves the pro-inflammatory cytokines, TNF-α, IL-1 and IL-6(162).

Other examples from which ACNs can mitigate brain damage caused by ROS are ACNs from Korean black beans that have been found to prevent hippocampal ethanol-induced ROS generation in postnatal mice. In addition, in the cortex of adult mice, ACNs from black soybean have been shown to prevent neuroinflammation and neurodegeneration induced by oxidative stress and an increase in ROS production (163,164).

Despite their numerous health benefits, some of the drawbacks of using ACNs are their limited bioavailability, instability at a physiological pH, and significant conversion into metabolites once absorbed, as well as by the gut flora. These drawbacks are the key factors limiting their therapeutic use and suggest that ACNs should primarily fulfill their function as direct scavengers in the gut (165-167).

In conclusion, ACNs are natural compounds with potent anti-inflammatory, antioxidant and neuroprotective properties. They are promising candidates for preventing chronic diseases and protecting against oxidative stress-induced damage. Nonetheless, addressing their limitations is critical for maximizing their potential therapeutic outcomes.

### Epigallocatechin gallate (EGCG)

Catechins present in green tea fall into two classes: Epistructured and non-epistructured catechins. The majority of the green tea catechins are from the epistructured catechins, primarily EGCG, then epicatechin gallate (ECG), epigallocatechin (EGC) and epicatechin (EC). The non-epistructured catechins are present in small quantities in green tea and constitute catechins gallate (CG), gallocatechin (GC), catechins (C) and gallocatechin gallate (GCG). *Camellia sinensis* (green tea) leaves synthesize the catechins through two metabolic pathways; the acetic-malonic and the shikimic-cinnamic acid (168,169).

EGCG has attracted significant attention for its several health benefits, particularly its neuroprotective effects, which are attributed to its antioxidant, radical scavenging, metal chelating, anti-carcinogenic, anti-apoptotic and anti-inflammatory characteristics. Research on EGCG has revealed its potential in supporting healthy aging by enhancing the morphological and functional changes that naturally occur in the aging brain, their capacity in inhibiting cognitive dysfunction, boosting learning capacity and minimize oxidative brain damage (170).

As previously demonstrated, whether administered before or after the beginning of EAE, EGCG reduced the clinical severity, brain inflammation and neuronal damage in animal models of MS and EAE. Scientists observed the reduced proliferation and TNF-α generation of encephalitogenic T-cells in mice following oral treatment. Furthermore, cell cycle arrest was observed in human myelin-specific CD^4+^ T-cells, which resulted in the downregulation of CDK4 upon treatment with EGCG (171). The suppression of the catalytic activity of the 20S/26S proteasome complex, which led to the intracellular accumulation of IκBα and the subsequent inhibition of NF-κB activation, was responsible for the interference with T-cell proliferation and effector function (171).

By the downregulation of NF-κB in T-cells, EGCG displays its anti-inflammatory effects and neuroprotective properties by preventing the generation of neurotoxic ROS in neurons. Moreover, as previously demonstrated, the simultaneous administration of EGCG and glatiramer acetate in the EAE model demonstrated synergistic benefits both *in vitro* and *in vivo* (171-173).

As compared to vitamins E and C, EGCG has been shown to be a more potent radical scavenger. Moreover, EGCG can chelate metals. To attenuate oxidative cell damage, its structure serves as a site of attachment for transition metals, neutralizing their activity by transforming them into a redox inactive complex (174-176).

Nonetheless, it is crucial to verify the ability of EGCG to cross the BBB before its involvement in neuroprotection is confirmed. As previously demonstrated, several flavonoids successfully diffused *in vitro* using brain endothelial cell lines co-cultured with astrocytes, and this was then further validated by *in vivo* research. The presence of EGCG in brain tissue samples was documented following the oral administration of the compound for 5 days (177-179).

The neuroprotective and neuro-rehabilitative properties of green tea polyphenols, particularly EGCG, have been well-established. EGCG has been demonstrated to enhance cell viability, decrease ROS generation, and increase apoptotic markers and endoplasmic reticulum stress markers. Moreover, EGCG has been shown to protect against glutamate excitotoxicity, 6-hydroxydopamine-induced toxicity, mitochondrial oxidative stress-induced apoptosis and mitochondrial malfunction (180-184). In addition, EGCG protects mitochondrial energetics, reduces brain inflammation and prevents neuronal damage, all of which help to delay the development of symptoms and improve a patient's outcomes in terms of cognitive function, learning capacity and lifespan. In addition to its neuroprotective properties, EGCG also has neuro-rescue activity as it encourages neurite development. Due to its potential as a potent disease-modifying drug with neuro-rescue and neuroprotective characteristics, EGCG is an effective potential candidate for the treatment of MS (171,172,185-190).

The effects of EGCG on T-cell function have also been investigated. To rule out any indirect effects from affecting accessory cells, a group of researchers performed a study to assess i) the effectiveness of EGCG supplementation *in vivo*; ii) the direct effects of EGCG on T-cell function using purified T-cells; and iii) the underlying mechanisms of EGCG-induced suppression on T-cells (191). According to the findings, EGCG reduced *ex vivo* T-cell proliferation when stimulated with an antiCD3/CD28 antibody. The findings demonstrated that *in vivo* amounts of dietary EGCG supplementation may be sufficient to inhibit T-cell proliferation (191).

Another study demonstrated that EGCG reduced the rate of the induced differentiation of naive CD^4+^ T-cells into pro-inflammatory Th1 cells, negatively affecting the expression of the primary signal transducer, phosphorylated signal transducer and activator of transcription (p-STAT)1, and the master regulator transcription factor T-bet. Moreover, EGCG reduced the naive CD4^+^ T-cell development into Th17 cells, which was accompanied by a decrease in their primary signal, p-STAT3(192). Nevertheless, EGCG did not affect the population of natural Tregs, while modestly increasing the frequency of induced Tregs (iTregs). Furthermore, EGCG significantly decreased the decline in iTreg differentiation caused by the injection of IL-6. These findings concluded that EGCG reduced Th1 and Th17 and enhanced the differentiation of iTregs (192).

Owing to the structure of EGCG, which suggests further antioxidant abilities, EGCG was found to be able to prevent neuronal damage induced by N-methyl-D-aspartate or TRAIL in living brain tissue, as well as directly obstruct the production of neurotoxic reactive oxygen species in neurons. By combining anti-inflammatory and neuroprotective properties, a natural green tea ingredient may provide a novel treatment option for young patients with inflammatory brain illness (171).

Overall, EGCG from green tea has immense potential as a natural and effective treatment option for a number of neurological conditions, including MS, due to its combined anti-inflammatory and neuroprotective properties. However, further research is required to fully comprehend its mechanisms and optimize its therapeutic use.

The aforementioned natural substances that are of potential benefit for the treatment of MS, molecular key targets and the model system used in studying them are presented in Table I.

## 5. Limitations

Limitations need to be carefully considered when exploring the use of naturally occurring molecules for the treatment of MS. Before applying these compounds to patients, two primary aspects demand thorough investigation: Safety and comparative efficacy compared to the existing medications. Furthermore, it is essential to investigate the safety of these molecules across diverse age and sex populations, as their effects may vary between different demographic groups. Additionally, considering the potential use of these compounds during lactation is of utmost importance to ensure the well-being of both the mother and the child.

By conducting well-controlled and comprehensive preclinical and clinical studies, a better understanding of the potential benefits and risks associated with these naturally occurring molecules can be obtained. Addressing these limitations may facilitate informed decision-making and may pave the way for the development of safer and more effective treatment options for patients with MS.

## 6. Conclusions and future perspectives

In summary, MS is a chronic progressive neurological disorder driven by various molecular pathways. While no approved cure currently exists, current medications aim to attenuate the disease progression without providing neuron protection. However, the presence of naturally occurring molecules that hold promise in alleviating MS symptoms and potentially offering neuroprotection warrant further investigation. Rigorous preclinical and clinical studies are essential to evaluate their potential efficacy, safety and effectiveness as standalone treatments or in combination with existing medications. By exploring these natural compounds, more effective and comprehensive treatment approaches may be developed for patients with MS, filling the gap left by current medication. Advancing research in this area holds the potential to significantly improve the lives of patients with MS and may lead to transformative therapeutic advancements in the future.

## Figures and Tables

**Table I:** A summary of the medicinal naturally occurring substances used for the treatment of MS, their molecular targets, the models implemented for studying their protective effects and their chemical structures.

Natural substance	Molecular targets	MS model (Refs.)	Molecular formula and/or structure description (Refs.)
Quercetin	IL-1β (4.0-fold), IL-6 (3.6-fold), IFN-γ (2.5-fold), TNF-α (2.0-fold) and IL-12p70 (5.7-fold) into the supernatants of DC-T cell co-cultures	*In vitro* model using dendritic cells (121-126,129)	C_15_H_10_O_7_; a pentahydroxy flavone (193)
Anthocyanin (ACNs)	TNF, IL-1, IL-6, NF-κB, CYP2E1, ROS, iNOS	Neuroprotective in mouse toxicological studies (150,151,154-156)	Flavylium cation backbone which is hydroxylated on different carbons (194)
Astaxanthin (AST)		*In vivo* rat model of CPZ-induced MS (110-114)	C_40_H_52_O_4_; 3,3'-dihydroxy- 4,4'-diketo-β-β carotene (195)
Epigallocatechin gallate (EGCG)	TNF-α, NF-κB, Th1, Th17, IL-6	Animal model of EAE (168-173)	C_22_H_18_O_11_; epigallocatechin- 3-gallate (196)

MS, multiple sclerosis; IL, interleukin; ROS, reactive oxygen species; iNOS, inducible nitric oxide synthase; EAE, experimental autoimmune encephalomyelitis; CPZ, cuprizone.

## Data Availability

Not applicable.
